# Profound Chemopreventative Effects of a Hydrogen Sulfide-Releasing NSAID in the APC^Min/+^ Mouse Model of Intestinal Tumorigenesis

**DOI:** 10.1371/journal.pone.0147289

**Published:** 2016-02-24

**Authors:** Mark Paul-Clark, Wagdi Elsheikh, Nicholas Kirkby, Melissa Chan, Pallavi Devchand, Terence A. Agbor, Kyle L. Flannigan, Charlotte Cheadle, Maxim Freydin, Angela Ianaro, Jane A. Mitchell, John L. Wallace

**Affiliations:** 1 Imperial College London, London, United Kingdom; 2 Department of Medicine, McMaster University, Hamilton, Ontario, Canada; 3 William Harvey Research Institute, London, United Kingdom; 4 Mount Sinai Hospital, Icahn Medical Institute, New York, United States of America; 5 Department of Experimental Pharmacology, Universita di Napoli Federico II, Napoli, Italy; 6 Department of Physiology & Pharmacology, University of Calgary, Calgary, Alberta, Canada; 7 Faculty of Medicine, Universidade Camilo Castelo Branco, São Paulo, SP, Brazil; CWRU/UH Digestive Health Institute, UNITED STATES

## Abstract

Nonsteroidal anti-inflammatory drugs have been shown to reduce the incidence of gastrointestinal cancers, but the propensity of these drugs to cause ulcers and bleeding limits their use. H_2_S has been shown to be a powerful cytoprotective and anti-inflammatory substance in the digestive system. This study explored the possibility that a H_2_S-releasing nonsteroidal anti-inflammatory drug (ATB-346) would be effective in a murine model of hereditary intestinal cancer (APC^Min+^ mouse) and investigated potential mechanisms of action via transcriptomics analysis. Daily treatment with ATB-346 was significantly more effective at preventing intestinal polyp formation than naproxen. Significant beneficial effects were seen with a treatment period of only 3–7 days, and reversal of existing polyps was observed in the colon. ATB-346, but not naproxen, significantly decreased expression of intestinal cancer-associated signaling molecules (cMyc, β-catenin). Transcriptomic analysis identified 20 genes that were up-regulated in APC^Min+^ mice, 18 of which were reduced to wild-type levels by one week of treatment with ATB-346. ATB-346 is a novel, gastrointestinal-sparing anti-inflammatory drug that potently and rapidly prevents and reverses the development of pre-cancerous lesions in a mouse model of hereditary intestinal tumorigenesis. These effects may be related to the combined effects of suppression of cyclooxygenase and release of H_2_S, and correction of most of the APC^Min+^-associated alterations in the transcriptome. ATB-346 may represent a promising agent for chemoprevention of tumorigenesis in the GI tract and elsewhere.

## Introduction

Although significant progress has been made in the detection, diagnosis and identification of specific molecular mechanisms of colorectal cancer, there is currently no cure for this disease [1]. The predominant form of hereditary cancer in the small and large intestine is known as Familial Associated Polyposis (FAP), which is mainly linked to defects in the Adenomatous Polyposis Coli (APC) gene. Moreover, seventy percent of sporadic colorectal cancers are due to bi-allelic inactivation of the APC gene. APC is a protein involved in the Wnt/β-catenin signaling pathway. Mutations in this signaling pathway are the only known genetic alterations present in early premalignant lesions in the intestine, such as aberrant crypt foci and small adenomas or polyps. Constitutive activation of the Wnt signaling pathway caused by mutations in components of the pathway has been suggested to be responsible for the initiation of colorectal cancer [2].

The experimental models used to study colorectal cancer largely involve use of animals with mutations in Wnt/β-catenin signaling pathway or by chemically stimulating alterations in these pathways to initiate tumorigenesis in the intestine. One of the most commonly used models is the heterozygous Apc^Min/+^ mouse [3]. This mouse is similar to human FAP in that it carries a mutation in the APC gene, predisposing them to develop multiple colonic and small intestinal polyps and adenomas.

There are extensive data suggesting that regular use of nonsteroidal anti-inflammatory drugs (NSAIDs) can markedly reduce the incidence of intestinal cancer [4,5]. Moreover, NSAIDs have been shown to have significant chemopreventative effects in numerous animal models of intestinal cancer [6,7]. The mechanism underlying the chemopreventative actions of NSAIDs are not clear, though suppression of prostaglandin E_2_ synthesis, particularly via inhibition of cyclooxygenase (COX)-2 activity, has been suggested to be important [8]. The major limitation to the widespread use of NSAIDs to reduce cancer risk in humans is the significant gastrointestinal (GI) adverse effects of these drugs. NSAIDs induce ulceration and bleeding throughout the GI tract, and such damage is more common in the elderly and in patients taking other anti-coagulants and with co-morbidities such as rheumatoid arthritis, hypertension and obesity [9,10]. Since the introduction of selective COX-2 inhibitors at the start of the 21^st^ century, physicians have become more aware of the significant risks of serious cardiovascular adverse effects of NSAIDs that further limit the use of this class of drugs for chemoprevention of cancer [11].

Hydrogen sulfide (H_2_S) is an endogenous signaling molecule with a wide range of anti-inflammatory, anti-oxidant and cytoprotective actions [12,13]. As well as directly scavenging reactive oxygen species [14,15] and inhibiting myeloperoxidase activity [16], H_2_S has been shown to production of several pro-inflammatory cytokines (by inhibiting nuclear factor kappa-light-chain-enhancer of activated B cells (Nf-KB) activity) [17], and to activate the Nrf2 (nuclear factor {erythroid-derived 2}-like 2)-regulated antioxidant response elements [18], possibly via protein S-sulfhydration [19]. H_2_S also exerts potent protective and reparative effects in the GI tract, some of which may be mediated, in part, through its anti-oxidant actions [20–23]. These powerful effects of H_2_S have been exploited in the development of several novel drugs [12]. For example, H_2_S-releasing derivatives of several NSAIDs have been developed with a primary aim of producing anti-inflammatory drugs with greatly reduced GI toxicity [12,20,24–26]. H_2_S-releasing NSAIDs have also been shown to exert significant beneficial effects in various rodent tumorigenesis models [27–30]. For example, an H_2_S-releasing derivative of the NSAID naproxen (ATB-346) produced significantly greater chemopreventative effects than equimolar doses of naproxen in the azoxymethane-induced tumorigenesis model in rats, without causing the significant GI injury caused by naproxen [30]. The suppression of GI prostaglandin synthesis by ATB-346 was comparable to that produced by naproxen, suggesting that effects other than suppression of COX activity accounted for the enhanced chemopreventative potency of this H_2_S-releasing drug [30].

In the present study, we have further evaluated the chemopreventative effects of ATB-346 versus naproxen, using the Apc^Min/+^ mouse model that closely mimics human FAP [3]. We have also attempted to identify potential mechanisms of action, including through a transcriptomics analysis of the effects of the two drugs.

## Materials and Methods

### Animals

Male C57BL/6 and Apc^Min/+^ mice were from Jackson Laboratories (Bar Harbor, MA, USA). All mice were housed in the Central Animal Facility at McMaster University. The mice were fed standard chow and water *ad libitum*, and were housed in a room with controlled temperature (22 ± 1°C), humidity (65–70%) and light cycle (12 h light/12 h dark). The Animal Care Committee of the Faculty of Health Sciences at McMaster University approved all experimental procedures. The studies were carried out in accordance with the guidelines of the Canadian Council of Animal Care. The health of the animals was assessed at least twice per day, and any animal in distress or having lost >15% of their original body weight was euthanized by an overdose of sodium pentobarbital.

### Onset of macroscopic polyp formation

To determine at what age Apc^Min/+^ mice began to exhibit macroscopically visible polyps, we euthanized groups of three Apc^Min/+^ mice and three wild-type mice at 5, 6, 8, 10, 12 and 14 weeks of age. The entire small intestine and colon was excised from each mouse, opened by a longitudinal incision, and blindly examined for polyps (see below). From this pilot study, we determined that polyps first appeared around week 12. All subsequent studies used week 14 as the endpoint.

### Calculation of Polyp Score

The mice were euthanized with isoflurane. The small intestine and colon were excised, opened longitudinally and the number and size (diameter) of polyps were blindly scored under a dissecting microscope, as described previously [30]. A separate count of the polyps for three regions of the intestine (duodenum, jejunum and ileum) and for the colon was recorded. A “total polyp score” was calculated for each mouse, which was the sum of the number of polyps for all areas. Samples of the intestine and colon were snap-frozen and stored at -80 C for subsequent Western blot analysis.

### Effects of treatment with ATB-346 and naproxen

Most studies involved treatment with a test drug or vehicle beginning when the Apc^Min/+^ mice had reached 6 weeks of age (n≥5 per group). The mice were treated orally once daily for between 3 and 14 days with vehicle (95:5, 1% CMC:DMSO), naproxen (1 or 10 mg/kg) or equimolar doses of ATB-346. In one experiment the effects of daily treatment with the H_2_S-releasing moiety of ATB-346 (TBZ) were assessed. In all experiments, the presence of intestinal and colonic polyps was assessed when the mice had reached 14 weeks of age.

### Western blot analysis

Western blot analysis was used to determine expression of two proteins linked to colon cancer, cMyc and β-catenin [31]. In week 14 of life, samples of colon 2 cm from the cecum were collected from Apc^Min/+^ mice that had treated during weeks 6 and 7 of life with ATB-346, naproxen or vehicle, and from vehicle-treated C57Bl/6 mice. The tissue was homogenized in ceramic bead tubes (1.8 mm tubes) (Mo Bio Laboratory Inc., Carlsbad, CA, USA) using Precellys 24 homogenizer (Bertin Technologies Corporations, Paris, France) for 3 intervals of 35 seconds at 6500 rpm. In a lysis buffer containing 50 mmol/l Tris (pH 8.0), 0.5% NP-40, 1 mmol/l EDTA, 150 mmol/l NaCl, 10% glycerol, 50 mmol/l sodium fluoride, 10 mmol/l sodium pyrophosphate, 1 mmol/l sodium orthovanadate, 1 mmol/l phenylmethylsulfonyl fluoride, and a tablet of protease inhibitor cocktail (Roche Diagnostics, Mannheim, Germany). Proteins were separated on 4–20% gradient polyacrylamide gels (Biorad, Mississauga, ON, Canada). Blots were incubated with blocking buffer (5% Bovine Serum Albumin Tris-buffered saline and Tween 0.05%) for 1 hour (Sigma, St. Louis, MO). They were then incubated in primary antibody overnight at 4°C. The blots were then washed with Tris-buffered saline containing 0.05% tween (3 x 10min). The blots were incubated (1 h, room temperature) with secondary anti-rabbit IgG antibody conjugated to horseradish peroxidase (1:1000). Enzymes were visualized using an enhanced chemiluminescence detection kit on a Chemi-doc gel imaging system (Bio-Rad). The intensity of the bands was determined and analyzed using ImageLab 2.0 software (Bio-Rad, Canada). The expression of each enzyme was normalized to the expression of β-actin (1:1000; Cell Signaling Technology, Beverly, MA, USA). Proteins were separated on 4–20% gradient polyacrylamide gels. Rabbit polyclonal cMyc (1:500) and β -catenin (1:500) were used (Cell Signaling Technology, Beverly, MA, USA). Enzyme expression was visualized using a secondary anti-rabbit IgG antibody conjugated to horseradish peroxidase (1:1000) and an enhanced chemiluminescence detection kit on a Chemi-doc gel imaging system (Bio-Rad, Canada). The intensity of the bands was determined and analyzed using ImageLab 2.0 software (Bio-Rad, Canada). The expression of each enzyme was normalized to the expression of β-actin (Cell Signaling Technology, Beverly, MA, USA, 1:1000).

### Transcriptomics analysis

Groups (n = 6–8) of 6-week old, male APC^Min/+^ mice were treated orally with vehicle (95:5, 1% CMC:DMSO), naproxen (10 mg/kg) or ATB-346 (equimolar dose) once daily for 7 days. A group of 6-week old, male C57BL/6 (wild-type) mice were treated on the same days with vehicle. Three hours after the final dose of the test drugs or vehicle, the mice were euthanized with isofluorane and a laparotomy was performed. A sample of colonic tissue (2 cm distal to the cecum) was collected and immediately snap-frozen. The samples were homogenized using Precellys 24 homogenizer (Bertin Technologies, Paris, France) and processed using an RNeasy Mini Kit in accordance with the manufacturer’s instructions (Qiagen, Toronto, Canada).

Total RNA extracted from colonic tissue was subject to standard microarray procedures. Purity and quality of RNA was assessed using an Agilent Bioanalyzer (Santa Clara, CA, USA). Samples were converted to labeled cDNA, fragmented and hybridized to Illumina MouseRef-8 v2.0 BeadChip arrays (Illumina, San Diego, CA, USA) by Source Bioscience (Nottingham, UK). Analysis of datasets was performed in GeneSpring (Agilent). Raw data were log_2_ transformed and then quantile-normalized. Pathway analysis of significantly modified genes between groups was performed in g:Profiler (http://biit.cs.ut.ee/gprofiler/). The transcriptomics data have been uploaded to the Gene Expression Omnibus, National Centre for Biotechnology Information.

### Statistical analysis

All data are expressed as the mean ± standard error of the mean (SEM). Comparisons among groups of data were performed by one-way analysis of variance followed by a post hoc test (Dunnett’s Multiple Comparison Test for parametric data and Mann Whitney Test for non-parametric data). In the case of the transcriptomics data, a one-way analysis of variance with Student-Neuman-Keuls post-hoc test and Storey’s bootstrapping multiple comparison correction were employed. With all analyses, an associated probability (p value) of less than 5% was considered significant.

### Materials

ATB-346 (2-(6-methoxy-napthalen-2-yl)-propionic acid 4-thiocarbamoyl-phenyl ester) was provided by Antibe Therapeutics Inc. (Toronto, ON, Canada). Naproxen sodium and diallyl disulfide were obtained from Sigma-Aldrich (Oakville, ON, Canada). Isoflurane was obtained from Abbott Laboratories (Montreal, Canada). TBZ (4-hydroxythiobenzamide) was purchased from SynChem Inc. (Des Plaines, IL, USA).

## Results

**Fig 1:**
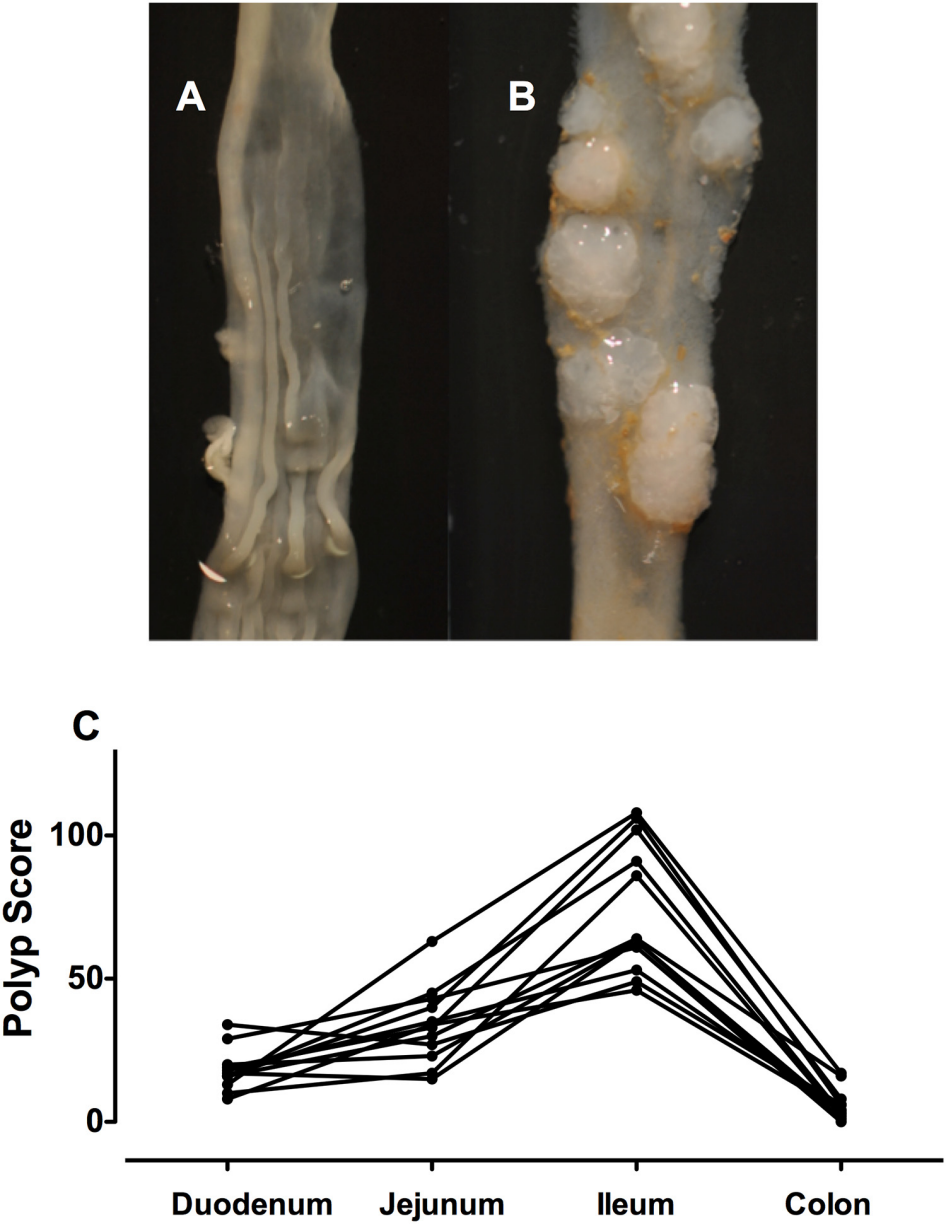
Polyp formation in the small intestine and colon of APC^Min+^ mice at 12 weeks of age. Panel A shows ileum from a wild-type (C57Bl/6) mouse, while panel B shows ileum from an APC^Min+^ mouse (with numerous polyps visible in the latter). Panel C shows the Polyp Score for 12 APC^Min+^ mice, illustrating the regional variation through the small intestine and colon. Each line represents the results from one mouse. The ileum exhibited the greatest polyp score, while the colon exhibited the lowest.

Significant numbers of polyps were apparent in the colon and small intestine by 12 weeks of age in the APC^Min/+^ mice. Based on this observation, we selected week 14 as the time for evaluation of the number of polyps in our experiments. None of the wild-type mice developed polyps (Fig 1A). In sharp contrast, all of the vehicle-treated APC^Min/+^ mice had polyps ranging in diameter from 1 to 4 mm (Fig 1B) and were found throughout the small intestine, but most predominantly in the terminal ileum (Fig 1C). The vehicle-treated APC^Min/+^ mice also presented with several colonic polyps that were generally larger in size than the intestinal polyps, ranging in diameter from 3 to 6 mm. These polyps were found throughout the colon, including the cecum.

### ATB-346 significantly inhibited polyp formation

**Fig 2:**
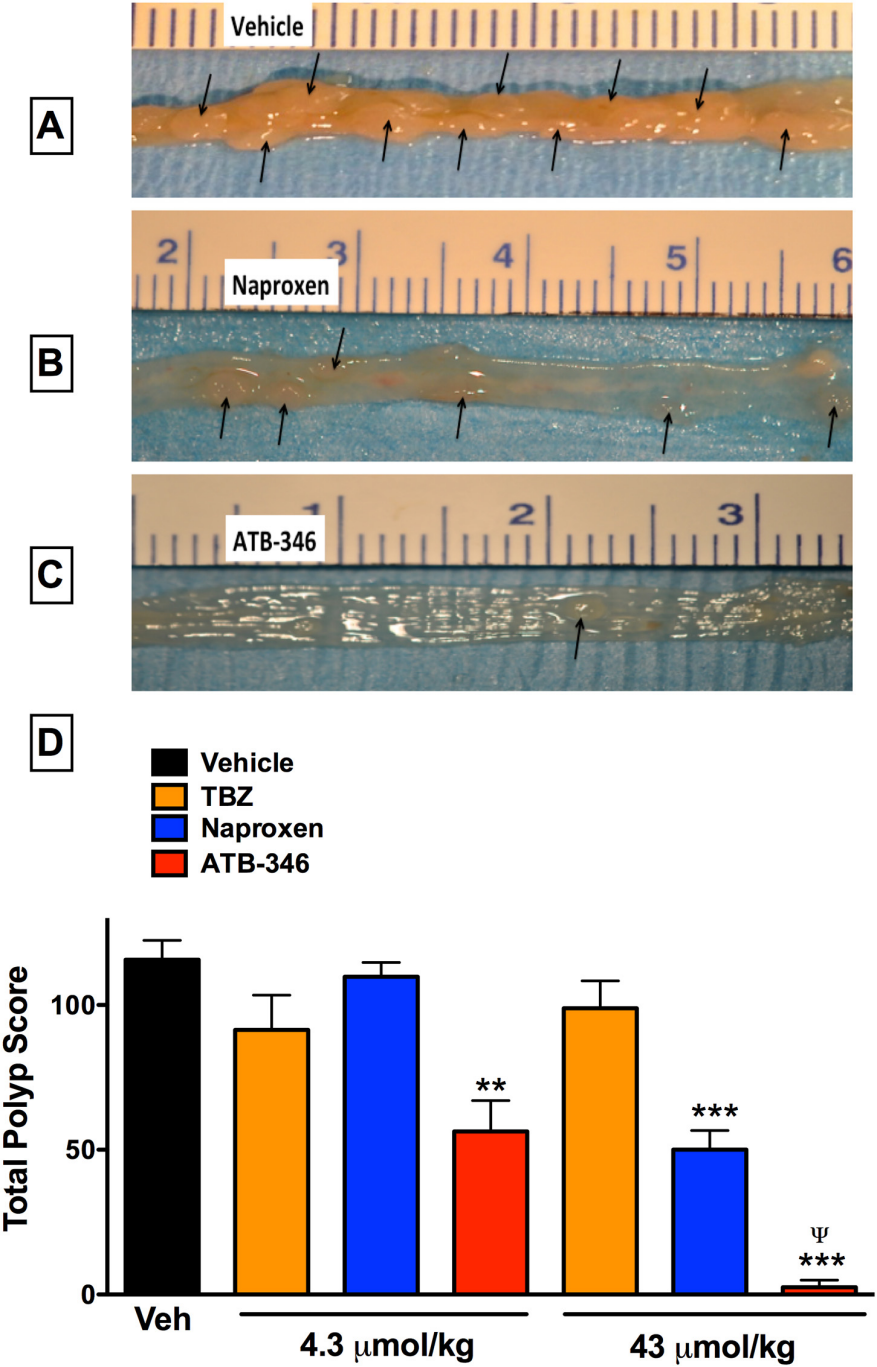
Dose-related preventative effects of oral treatment with ATB-346 or naproxen on intestinal polyp formation in APC^Min/+^ mice. Groups of at least 6 mice each were treated daily with vehicle, naproxen (1 or 10 mg/kg), or equimolar doses of ATB-346 or of the hydrogen sulfide-releasing moiety of ATB-346 (TBZ; 4-hydroxythiobenzamide). Treatments were started at week 6 of life, and the numbers and areas (in mm^2^) of polyps in the small intestine and colon were blindly assessed at week 14 of life (panels A, B and C show examples; arrows indicate polyps). Panel D shows the ‘total polyp score’ data (the sum of the areas of all polyps in each mouse). Data are shown as the mean ± SEM. **p<0.01, ***p<0.001 versus the vehicle-treated group (one-way ANOVA and Dunnett’s test). ^Ψ^p<0.05 versus the group treated with naproxen at the same dose (Student’s t-test).

APC^Min/+^ mice treated daily for 14 days (during the 6^th^ and 7^th^ weeks of life) with naproxen at a dose of 1 mg/kg (4.3 μmol/kg) had similar numbers of polyps as vehicle-treated APC^Min/+^ mice (Fig 2). In contrast, daily treatment with ATB-346 at an equimolar dose resulted in 51% reduction (p<0.01) in the total polyp score. With a higher dose of naproxen (10 mg/kg; 43 μmol/kg), a marked reduction (57%; p<0.001) of the total polyp score was observed. However, treatment with an equimolar dose of ATB-346 reduced the total polyp score by 98%, significantly greater than the effect of naproxen. Daily treatment for 14 days with 4-hydroxy-thiobenzamide (TBZ), the H_2_S-releasing moiety of ATB-346, did not significantly affect the total polyp score at either dose.

**Fig 3:**
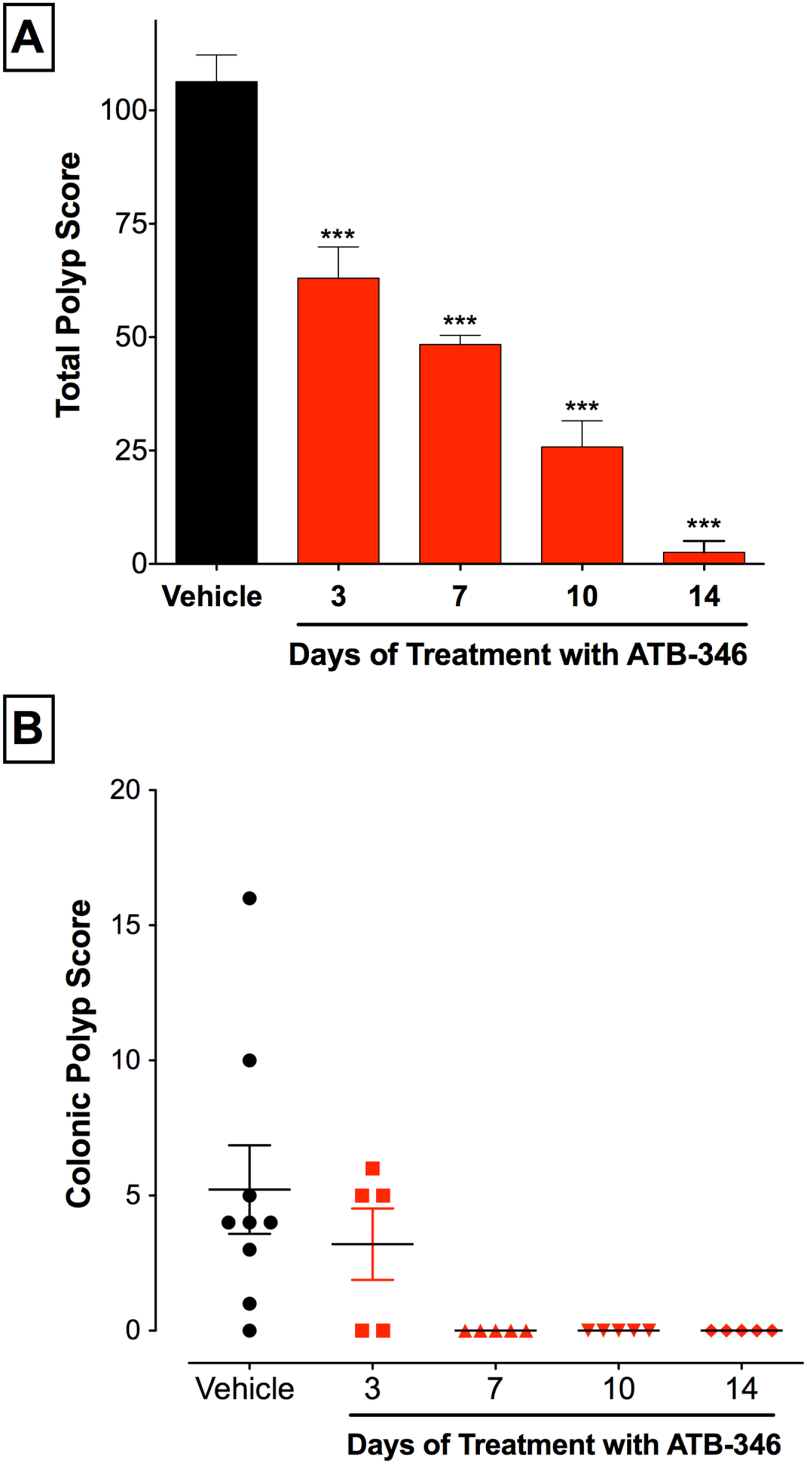
Duration-of-treatment-dependent reduction of total polyp score by ATB-346. Panel A: APC^Min/+^ mice treated with ATB-346 at 14.5 mg/kg (equimolar to 10 mg/kg of naproxen) for up to 14 days, starting at week 6 of life, had significantly lower total polyp scores as compared to vehicle-treated mice (***p<0.001 compared to the vehicle-treated group). Data are expressed as mean ± SEM (n≥5 per group). Panel B: Apc^Min/+^ mice treated once daily with ATB-346 for up to 14 days had significantly lower colonic polyp scores compared to vehicle. In the 7-, 10- and 14-day treatment groups, there was a complete absence of polyps (p<0.05; Mann-Whitney test; n≥5 per group). The bars on the figure represent the mean ± SEM.

The magnitude of reduction of polyp formation in APC^Min/+^ mice treated with ATB-346 increased with the duration of treatment (Fig 3A). A significant reduction of polyp formation was observed with only 3 days of treatment (~40%; p<0.001), and progressively greater reductions were seen with 7, 10 and 14 days of treatment, the latter producing almost complete prevention of polyp formation.

**Fig 4:**
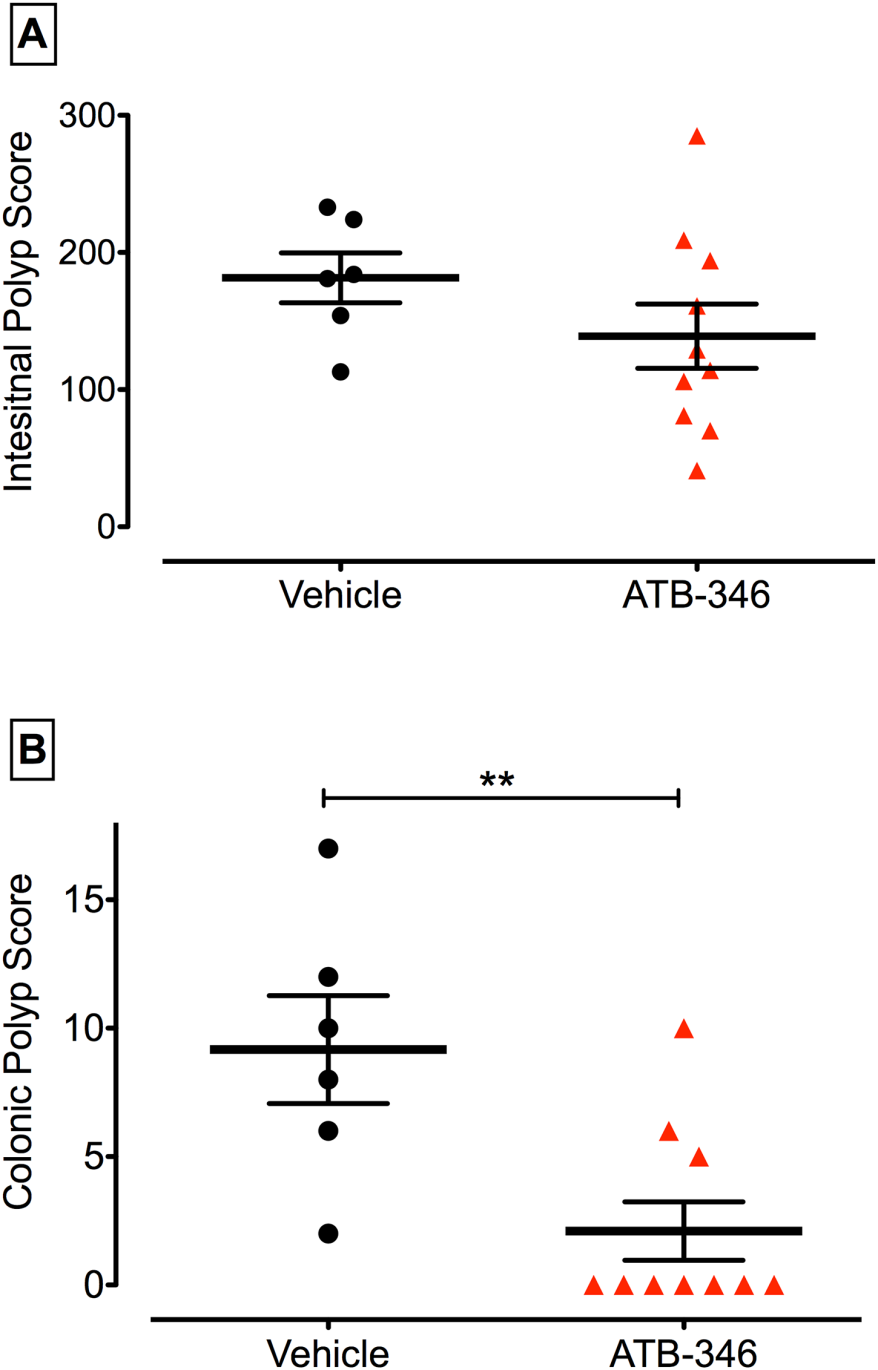
Colon-specific reduction/reversal of polyps. Initiation of treatment with ATB-346 (14.5 mg/kg once daily for 14 days) at week 12 had no effect on the small intestinal polyp score (assessed at week 14), but markedly reduced polyp formation in the colon (**p<0.01).

As shown in Fig 1, the number of polyps developing in the colon was lower than in the ileum. However, the ability of ATB-346 to prevent colonic polyp formation was more rapid in onset (Fig 4B). Colonic polyp formation was completely prevented by daily treatment with ATB-346 (14.5 mg/kg) for 7 or more days.

### Treatment with naproxen or ATB-346 for two weeks beginning at 12 weeks of age

Daily treatment of Apc^Min/+^ mice with ATB-346 (14.5 mg/kg) beginning at 12 weeks of age, and continuing for 14 days, did not significantly alter the number or intestinal polyps (Fig 4). In contrast, there was a marked reduction in the number of colonic polyps that were evident at the end of the study (p<0.01 versus vehicle-treated). Treatment with naproxen (10 mg/kg) following the same protocol did not produce a significant reduction of polyp numbers in the intestine or colon.

### ATB-346 normalized β–catenin and cMyc expression in APC^Min/+^ mice

**Fig 5:**
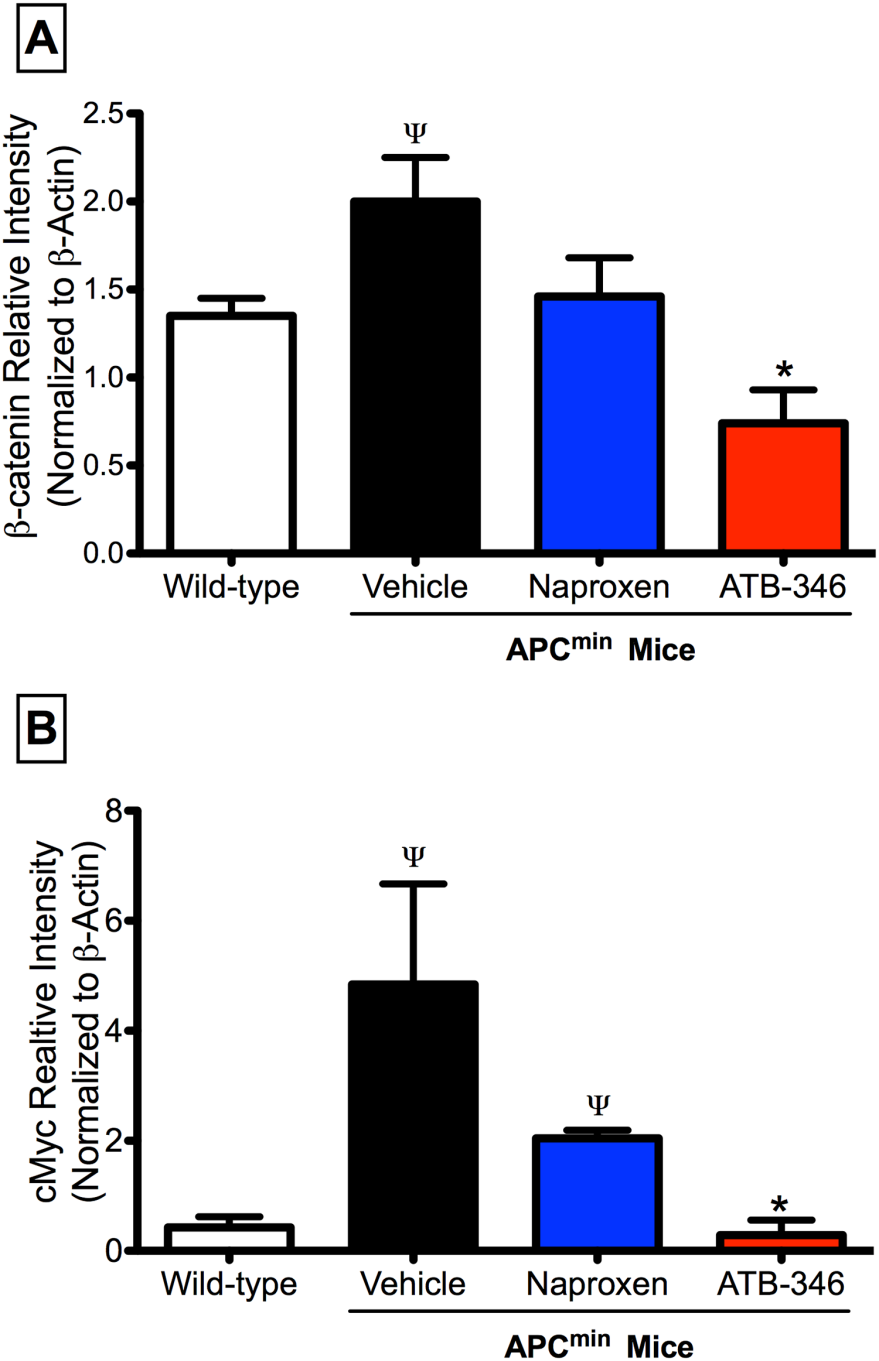
Treatment with ATB-346 reduced intestinal β-catenin and cMyc levels in APC^Min/+^ mice. Expression of both β-catenin (panel A) and cMyc (panel B) were significantly elevated in colonic tissue from APC^Min/+^ mice as compared to wild-type mice. Treatment of APC^Min/+^ mice with ATB-346 (14.5 mg/kg) significantly reduced β-catenin and cMyc expression to levels comparable to those in wild-type mice, while naproxen treatment (10 mg/kg) reduced cMyc but not β-catenin. Tissue samples were collected from the mice at 14 weeks of age, while drug treatment occurred in the 6^th^ and 7^th^ weeks of age. Each bar represents the mean ± SEM for at least 4 mice (p<0.05 vs. wild-type mice; ^ψ^p<0.05 vs. wild type. *p<0.05 vs. naproxen-treated).

Expression of the well-established markers of APC-associated tumorigenesis, β–catenin and cMyc, was significantly elevated in samples of colon from APC^Min/+^ mice as compared to wild type mice (Fig 5). Treatment with ATB-346 (14.5 mg/kg) for 7 days normalized expression of both markers to a significantly greater extent than treatment with an equimolar dose (10 mg/kg) of naproxen.

### Differential effects of naproxen versus ATB-346 on the transcriptome in the colon of APC^Min/+^ mice

To determine the influence of naproxen and ATB-346 on expression of genes associated with polyp formation, a transcriptome-wide analysis of colon tissue from APC^Min/+^ mice, treated with naproxen (10 mg/kg), ATB-346 (14.5 mg/kg) or vehicle for 7 days (between 6 and 7 weeks of age) was conducted. As a comparator, tissue from vehicle-treated wild-type (C57Bl/6) mice was also studied to determine the effect of the APC^Min/+^ mutation on the transcriptome of the colonic tissue.

Box and whisker plots of all raw intensity values showed little variation among samples from all groups analyzed. Principle component analysis showed minimal clustering of global patterns, which likely reflects relatively modest changes in the transcriptome among the groups (data not shown).

**Fig 6:**
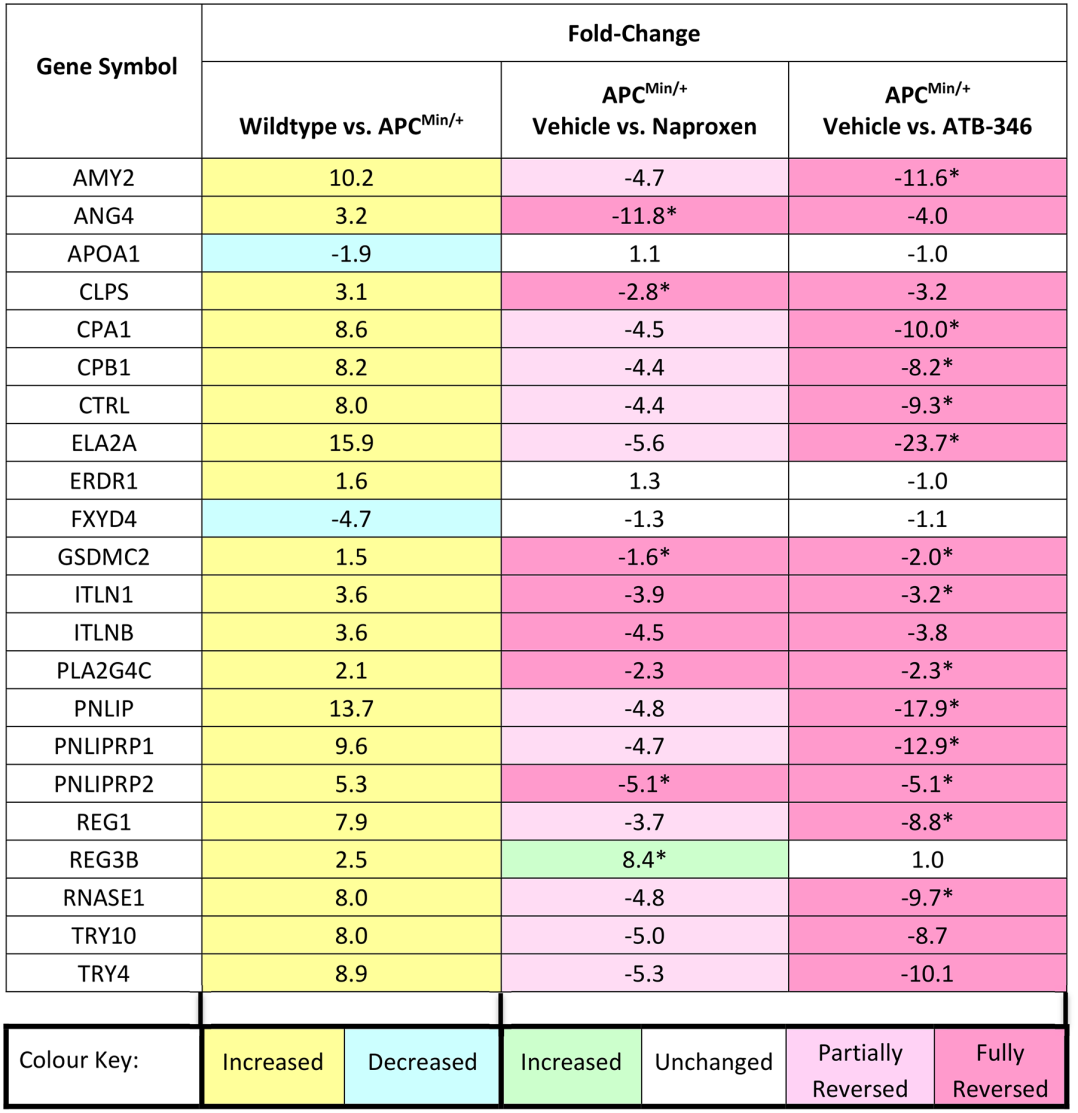
Summary of transcriptomics analysis. Genes altered by >1.5-fold in the colon of APC^Min/+^ mice as compared to wild-type mice, and the effects of daily treatment for one week with naproxen or ATB-346. Fold-change values represent direction of change from the first listed condition to the second. During the 6^th^ week of age, mice were treated with vehicle, naproxen at a dose of 10 mg/kg, or ATB-346 at an equimolar dose. Tissue samples were harvested at the start of the 7^th^ week of age. *p<0.05 by one-way ANOVA with Student-Neuman-Keuls post-hoc test and Storey’s bootstrapping multiple comparison correction. n = 6-8/group.

In the present study we identified 20 genes with ≥1.5-fold increases colonic expression related to the APC^Min/+^ mutation (Fig 6). Treatment with ATB-346 resulted in a reduction of expression of 18 of these genes to the levels observed in wild-type mice. Treatment with naproxen resulted in a comparable reduction in expression of 7 genes to that achieved with ATB-346, and the expression of a further 11 genes was partially reduced by naproxen (not achieving statistical significance in most cases). There were two genes with significantly reduced expression related to the APC^Min/+^ mutation (versus wild-type), neither of which was affected by treatment with ATB-346 or naproxen (Fig 6). One gene (*Reg3b*) was markedly up-regulated by naproxen, but not by ATB-346.

Pathway analysis revealed that the affected genes included some implicated in metabolism of phospholipids (GO:0006644) and lipids (GO:0006629), as well as hydrolase activity (GO:0016787). Enriched network analysis of pathway interactions showed a hub around *Apoa1*, which encodes for apolipoprotein A1. There were no significant associations to recognized cancer-associated networks of the genes altered by ATB-346 or by naproxen. However, two of the genes with increased expression in APC^Min/+^ mice, *Pnliprp1 and Pnliprp2*, are regulated by Runx1 [32], which is known to be an Nrf2-controlled gene [33]. Nrf2 can protect tissues from a variety of carcinogens by increasing the expression of a number of cytoprotective genes, and Nrf2 can be activated by H_2_S [33,34].

## Discussion

The US National Cancer Institute has predicted that there will be over 130,000 new cases of colon cancer in the USA in 2015, and almost 50,000 colon cancer-related deaths [1]. Chemoprevention strategies for GI cancers have been pursued for decades, particularly focused on the use of aspirin and other NSAIDs [4,5]. However, the propensity of COX inhibitors to cause bleeding in the GI tract and elsewhere has been the major limitation to this approach [9,10]. In the present study, we have demonstrated that a novel type of NSAID derivative, which does not produce GI damage at anti-inflammatory doses [25,35], is also substantially more effective than the parent NSAID (naproxen) in preventing the formation of polyps in a mouse model that closely mimics heritable intestinal polyposis in humans [3]. The reduced GI-damaging effects of ATB-346 (and other H_2_S-releasing NSAIDs) [24–26] is attributable to its ability to release small amounts of H_2_S [36], which has been shown to have potent anti-oxidant, cytoprotective and anti-inflammatory effects in the GI tract and in other tissues [12,13–23].

In the present study, ATB-346 produced significant beneficial effects in the APC^Min+^ mouse model when given for a period as short as 3 days, and was effective in the colon even when administered after polyps had already formed. ATB-346 was significantly more potent than naproxen in reducing polyp formation, despite inhibiting COX activity in the GI tract to the same extent [25,30,36]. These effects of ATB-346 were accompanied by a normalization of tissue expression of two well-characterized cancer markers, β-catenin and cMyc, with significantly greater effects than an equimolar dose of naproxen. Moreover, treatment with ATB-346 reduced the expression (to wild-type levels) of 18 of the 20 genes that were over-expressed in APC^Min+^ mice.

The enhanced effectiveness of ATB-346 in reducing polyp formation in APCMin+ mice is likely due to the H_2_S released from this drug. In support of this statement, there are a number of reports that H_2_S donors and NSAID-H_2_S conjugates reduced the incidence and/or severity of cancer in a range of animal models and cell lines, including cell lines that do not express COX [27–30,37]. On the other hand, administration of TBZ (the H_2_S-releasing moiety of ATB-346) did not significantly affect the polyp score at either dose tested (see Fig 2). However, this may be a consequence of reduced generation of H_2_S from the compound when administered alone, as compared to that from the ATB-346 conjugate. We have previously observed that the amount of H_2_S released from TBZ when it is bound to naproxen (i.e., ATB-346) is 4-fold greater than the release detected from the TBZ molecule alone [38].

The transcriptomic analysis of ~25,000 genes revealed that there were 20 genes that were up-regulated ≥1.5-fold in the colon of APC^Min+^ versus wild-type mice, and treatment with ATB-346 fully reduced expression of 18 (90%) of those genes to normal levels. Naproxen only fully reduced expression of 7 of the genes, and partially reduced expression of 11 genes. These findings suggest that the normalization of gene expression in the colon by ATB-346 may have occurred in part because of effects on COX activity, but other actions of the drug must have also contributed, since ATB-346 and naproxen inhibit COX to a comparable extent at equimolar doses. Again, the release of H_2_S from ATB-346 is the most likely explanation for these differences. COX-2 is a key oncogene in a number of human cancers. Celecoxib (a selective COX-2 inhibitor) was being developed as a preventative therapy for colon cancer until cardiovascular safety concerns arose that limited its use for such applications [39]. In addition, all NSAIDs, including those with selectivity for COX-2, can cause significant damage and bleeding throughout the gastrointestinal tract [9,10], and this is a major limitation to use of drugs like aspirin and naproxen for long-term chemoprevention of cancer.

The gene most profoundly down-regulated by ATB-346 was *Ela2a* (elastase 2), which encodes for the proteasome chymotrypsin-like enzymes. Although they have not been directly associated with gastrointestinal cancers, these enzymes are likely to be involved in tumor invasiveness, due to their matrix degradation properties [40]. This hypothesis is supported by the fact that proteasome inhibitors have shown therapeutic promise in a variety of tumor types [41]. It is noteworthy that matrix degradation can be modulated by H_2_S [42]. Other cancer-associated genes of interest that were profoundly down-regulated by ATB-346 (and less so by naproxen) included: (i) *Reg1*, which is up-regulated in human colorectal cancer [43] and is a predictor of cancer-associated death [44]; (ii) *Cpa1* and *Cpb1*, which are induced in gastrointestinal tumors [45] (iii) *Amy2*, which is up-regulated in a number of cancers including colonic neoplasms [46] and (iv) *Pnliprp1*, which has been identified in non-dissected tumors and micro-dissected invasive tumor cells [47]. The contribution of these ATB-346-sensitive genes to colon cancer remains the subject of investigation. However, it was striking that many of the genes down-regulated by ATB-346 were those that were strongly up-regulated in the APC^Min/+^ colon compared to wild-type, suggesting that ATB-346 can reverse much of the pre-cancerous gene expression profile in the colon in this model. A full and detailed validation of these genes may lead to the identification of novel pathways that not only reveal the mechanism underlying the COX-independent actions of ATB-346, but also novel targets for future drug development.

The transcriptomics analysis identified an unusual and specific effect of treatment with naproxen. Naproxen-treated APC^Min+^ mice exhibited a >8-fold increase in expression of the *Reg3b* gene as compared to vehicle-treated APC^Min+^ mice (21-fold greater than expression in wild-type mice). *Reg3b* is expressed in mouse intestine [48], and it encodes murine orthologues of human pancreatitis-associated proteins. These proteins are involved in the innate immune response to bacterial colonization of the intestinal tract [49]. The reason that *Reg3b* gene was up-regulated specifically in naproxen-treated is likely a consequence of the well-characterized damaging effects of naproxen in the intestine, and the ensuing bacterial colonization of the damaged regions [50,51]. Up-regulation of *Reg3b* has been reported in murine models of colitis [49]. The lack of a similar up-regulation of *Reg3b* in ATB-346-treated APC^Min+^ mice is consistent with the GI-safe profile of this drug [25,36].

ATB-346 is a novel anti-inflammatory drug that combines COX inhibition and release of H_2_S. The substantial increase in GI safety of this drug as compared to conventional NSAIDs should make it an attractive option for various therapeutic applications. We observed a more profound beneficial effect of ATB-346 in the colon than in the small intestine, but the reasons for this difference are not yet clear. The results of the present study suggest that ATB-346 may be a particularly useful compound for chemoprevention, and possibly reversal, of cancers in the GI tract.

## Abbreviations

ANOVA: analysis of variance
APC: adenomatous polyposis coli
COX: cyclooxygenase
FAP: familial adenomatous polyposis
GI: gastrointestinal
NF-κB: nuclear factor kappa-light-chain-enhancer of activated B cells
Nrf2: nuclear factor (erythroid-derived 2)-like 2
NSAID: nonsteroidal anti-inflammatory drug
PG: prostaglandin
SEM: standard error of the mean
TBZ: 4-hydroxy-thiobenzamide
